# Association of Sex, Age, and Eastern Cooperative Oncology Group Performance Status With Survival Benefit of Cancer Immunotherapy in Randomized Clinical Trials: A Systematic Review and Meta-analysis

**DOI:** 10.1001/jamanetworkopen.2020.12534

**Published:** 2020-08-07

**Authors:** Fang Yang, Svetomir N. Markovic, Julian R. Molina, Thorvardur R. Halfdanarson, Lance C. Pagliaro, Ashish V. Chintakuntlawar, Rutian Li, Jia Wei, Lifeng Wang, Baorui Liu, Grzegorz S. Nowakowski, Michael L. Wang, Yucai Wang

**Affiliations:** 1 The Comprehensive Cancer Center of Drum Tower Hospital, Clinical Cancer Institute of Nanjing University, Nanjing, China; 2 Division of Medical Oncology, Mayo Clinic, Rochester, Minnesota; 3 Division of Hematology, Mayo Clinic, Rochester, Minnesota; 4 Department of Lymphoma/Myeloma, The University of Texas MD Anderson Cancer Center, Houston

## Abstract

**Importance:**

Sex, age, and Eastern Cooperative Oncology Group (ECOG) performance status (PS) may affect immune response. However, the association of these factors with the survival benefit of cancer immunotherapy with immune checkpoint inhibitors (ICIs) remains unclear.

**Objective:**

To assess the potential sex, age, and ECOG PS differences of immunotherapy survival benefit in patients with advanced cancer.

**Data Sources:**

PubMed, Web of Science, Embase, and Scopus were searched from inception to August 31, 2019.

**Study Selection:**

Published randomized clinical trials comparing overall survival (OS) in patients with advanced cancer treated with ICI immunotherapy vs non-ICI control therapy were included.

**Data Extraction and Synthesis:**

Pooled OS hazard ratio (HR) and 95% CI for patients of different sex, age (<65 and ≥65 years) or ECOG PS (0 and ≥1) were calculated separately using a random-effects model, and the heterogeneity between paired estimates was assessed using an interaction test by pooling study-specific interaction HRs. This study followed the Preferred Reporting Items for Systematic Reviews and Meta-analyses (PRISMA) guideline.

**Main Outcomes and Measures:**

The difference in survival benefit of ICIs between sex, age (<65 vs ≥65 years), and ECOG PS (0 vs ≥1), as well as the difference stratified by cancer type, line of therapy, agent of immunotherapy, and immunotherapy strategy in the intervention arm.

**Results:**

Thirty-seven phase 2 or 3 randomized clinical trials involving 23 760 patients were included. An OS benefit of immunotherapy was found for both men (HR, 0.75; 95% CI, 0.71-0.81) and women (HR, 0.79; 95% CI, 0.72-0.88); for both younger (<65 years: HR, 0.77; 95% CI, 0.71-0.83) and older (≥65 years: HR, 0.78; 95% CI, 0.72-0.84) patients; and for both patients with ECOG PS 0 (HR, 0.81; 95% CI, 0.73-0.90) and PS greater than or equal to 1 (HR, 0.79; 95% CI, 0.74-0.84). No significant difference of relative benefit from immunotherapy over control therapy was found in patients of different sex (*P* = .25, *I^2^* = 19.02%), age (*P* = .94, *I^2^* = 15.57%), or ECOG PS (*P* = .74, *I^2^* = 0%). No significant difference was found in subgroup analyses by cancer type, line of therapy, agent of immunotherapy, and immunotherapy strategy in the intervention arm.

**Conclusions and Relevance:**

This meta-analysis found no evidence of an association of sex, age (<65 vs ≥65 years), or ECOG PS (0 vs ≥1) with cancer immunotherapy survival benefit. This finding suggests that the use of ICIs in advanced cancer should not be restricted to certain patients in sex, age, or ECOG PS categories.

## Key Points

**Question:**

Are different sex, age, and Eastern Cooperative Oncology Group performance status (0 vs ≥1) factors associated with the same benefit with immune checkpoint inhibitor immunotherapy compared with non–immune checkpoint inhibitor control therapy in the treatment of advanced cancers?

**Findings:**

In this meta-analysis of 37 randomized clinical trials involving 23 760 patients, no evidence of a significant difference in overall survival benefit from immune checkpoint inhibitors over control therapy between patients with different sex, age, or Eastern Cooperative Oncology Group performance status was found.

**Meaning:**

The results of this meta-analysis suggest that immunotherapy may confer a survival benefit in the treatment of advanced cancer regardless of patient sex, age, and performance status and should not be restricted based on these characteristics.

## Teaser

This meta-analysis examines the association of sex, age, and Eastern Cooperative Oncology Group performance status with overall survival in patients treated with immune checkpoint inhibitors for advanced cancer.

## Introduction

Immune checkpoint inhibitors (ICIs) have demonstrated efficacy against various hematologic and solid cancers.^1^ Because the biological basis of ICIs is to enhance antitumor immunity, patients who differ in immunologic responses may achieve different benefit from ICIs. Sex is a well-known variable that can potentially affect immune responses. Generally, women mount stronger innate and adaptive immune responses than men, which result in faster clearance of pathogens and greater vaccine efficacy.^2^ Moreover, the immune system experiences major changes with aging, when substantial immune cells become altered^3,4,5,6^ and adaptive immunity becomes less functional.^7,8^ Apart from sex and age, one study also showed that markedly altered Eastern Cooperative Oncology Group performance status (ECOG PS) was associated with worse immune response.^9^ Considering the differences in immune systems among patients, it is reasonable to postulate that the responses to immunotherapy may vary according to patient sex, age, and ECOG PS.

Sex-associated differences in survival benefit have been recently examined by Conforti et al,^10^ who demonstrated that men derived greater benefit from cancer immunotherapy compared with women. Conflicting results were reported by Wallis et al,^11^ who found no statistically significant association of patient sex with the magnitude of benefit from immunotherapy in advanced cancers. The correlations between patients’ age and cancer immunotherapy efficacy have been assessed by Wu et al,^12^ who reported that patients aged 65 years or older benefited more from immunotherapy than younger patients. In contrast, Kasherman et al^13^ suggested that ICIs improved overall survival (OS) for both younger and older patients, and the magnitude of OS improvement was age independent. To our knowledge, no study has assessed the association of ECOG PS with the relative benefit from immunotherapy in patients with advanced cancer.

Given the conflicting results regarding sex and age association with immunotherapy benefit and the lack of study of the association of ECOG with PS immunotherapy outcome, we performed a meta-analysis to examine the potential association of sex, age, and ECOG PS with immunotherapy survival benefit in patients with advanced cancer. We limited our study to randomized clinical trials that compared immunotherapy with ICIs and control therapy without ICIs and included several new randomized clinical trials that were published after previous systematic reviews.^10,11,12,13^

## Methods

For this meta-analysis, we conducted a literature search to identify randomized clinical trials comparing OS in patients with advanced cancer treated with immunotherapy with ICIs vs control therapy without ICIs. This study was registered with PROSPERO. The need for institutional review board approval was waived by Drum Tower Hospital because this study does not involve direct human subject research. We performed the study in adherence with the Preferred Reporting Items for Systematic Reviews and Meta-analyses (PRISMA) guidelines.^14^

### Study Selection

We searched PubMed MEDLINE, Web of Science, Embase, and Scopus from inception to August 31, 2019, to identify phase 2 or 3 randomized clinical trials of cancer therapy with ICIs. Two investigators (F.Y. and Y.W.) conducted independent searches using the terms *CTLA-4*, *cytotoxic T-lymphocyte–associated protein 4*, *PD-1*, *programmed death receptor 1*, *PD-L1*, *programmed cell death ligand 1*, *immune checkpoint inhibitor*, *ipilimumab*, *tremelimumab*, *nivolumab*, *pembrolizumab*, *atezolizumab*, *durvalumab*, and *avelumab*. References from the included studies were also reviewed to identify additional eligible studies.

For inclusion, studies had to meet all of the following criteria: (1) cancer therapy clinical trials using a randomized controlled design; (2) participants in the intervention group treated with a single ICI or ICI combinations, and participants in the control group received therapies without ICIs; (3) data available for the hazard ratio (HR) for death according to patients’ sex, age, or ECOG PS; and (4) published in English. If multiple reports of a given study were available, the one with the most updated and/or comprehensive data was included in this analysis and the duplicates were excluded.

Data from each study were extracted independently by 2 of us (F.Y. and Y.W.). Disagreements were resolved by consensus. We extracted study characteristics, including year of publication, first author, journal, trial name, phase, National Clinical Trial number, cancer type, line of therapy, and treatment arms. In addition, outcome information, including HR with 95% CI for death stratified by patient sex, age, and/or ECOG PS, was collected.

### Statistical Analysis

To assess the OS benefit from immunotherapy with ICIs, random-effects models were used to calculate the pooled HRs of death (ICI therapy vs control therapy) in paired groups, ie, men vs women, younger (<65 years) vs older (≥65 years), and patients with ECOG PS 0 vs ECOG PS 1 and above.

To assess the potential differences of survival benefit of ICIs between different sex, age, or ECOG PS groups, we first calculated a study-specific interaction HR (95% CI) in each study based on the reported HRs (95% CIs) in paired groups and then combined the study-specific interaction HRs across trials, using a random-effects model, to generate a *P* value for heterogeneity as described previously by Conforti et al^10^ and Wallis et al.^11^ The null hypothesis is that the survival benefit of ICIs is equal between the paired groups, and a *P* value for heterogeneity <.05 was considered to indicate a statistically significant difference of the relative survival benefits between the groups.

We performed subgroup analyses to explore the variation of the effect of sex, age, and ECOG PS on immunotherapy survival benefit. The subgroups included cancer type, line of therapy, agent of immunotherapy, and immunotherapy strategy in the intervention arm.

We identified between-study heterogeneity using the *Q* test and calculated the *I*^2^ values. The *I*^2^ statistic was used to quantify heterogeneity among the studies, and degrees of heterogeneity were considered low for *I*^2^ values of 25%, moderate for 50%, and high for 75%.^15^

All reported *P* values are 2-sided, and a *P* value <.05 was considered to indicate statistical significance. We conducted all analyses using Comprehensive Meta-Analysis, version 2 (Biostat Inc).

## Results

We screened a total of 43 376 records, of which 158 were reviewed in full. In total, 37 studies were included for analysis involving 23 760 patients with advanced cancers (Figure). The characteristics of the included studies^16,17,18,19,20,21,22,23,24,25,26,27,28,29,30,31,32,33,34,35,36,37,38,39,40,41,42,43,44,45,46,47,48,49,50,51,52^ are summarized in the eTable in the Supplement. Most of the trials were phase 3 (n = 34) and conducted for subsequent lines of therapy (n = 22). The most common cancer types were non–small cell lung cancer (n = 14) and melanoma (n = 5). The most common ICIs used were anti-programmed cell death receptor 1 (PD-1)/programmed cell death receptor ligand 1 (PD-L1) inhibitors (n = 27).

**Figure.**
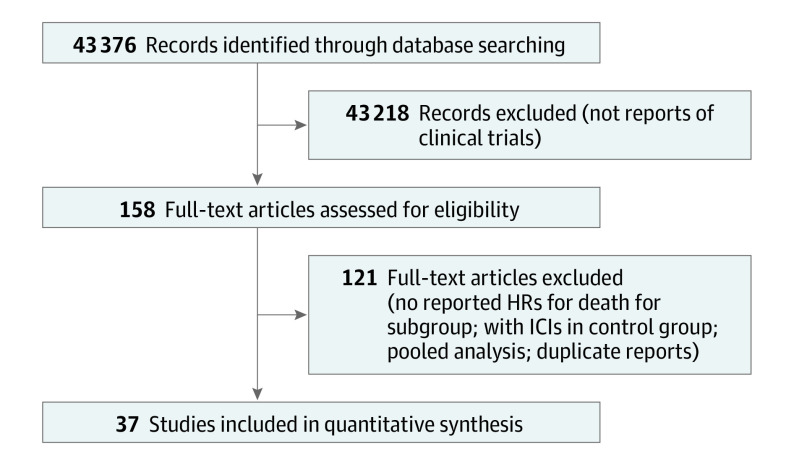
Study Selection Process HRs indicates hazard ratios; ICIs, immune checkpoint inhibitors.

A total of 32 trials enrolling 20 699 patients reported data on HR for death according to patients’ sex; 13 674 were men (66.1%) and 7025 were women (33.9%). An OS advantage of immunotherapy compared with control therapy was observed for both men (HR, 0.75; 95% CI, 0.71-0.81) and women (HR, 0.79; 95% CI, 0.72-0.88) (eFigure 1 in the Supplement). There was no significant difference in OS from ICIs over control therapy between men and women (*P* = .25, *I^2^* = 19.02%) (eFigure 2 in the Supplement; Table 1). Statistically significant heterogeneity was found among the studies for both men (*Q* = 69.31, *P* < .001, *I^2^* = 52.39%) and women (*Q* = 75.18, *P* < .001, *I^2^* = 56.11%). There was no significant difference found in subgroup analyses by cancer type, line of therapy, agent of immunotherapy, or intervention therapy strategy (Table 1).

**Table 1.** Differences in Survival Benefit Associated With Immunotherapy in Men and Women by Subgroups

Variable	Studies, No.	Participants, No.		Pooled HR (95% CI) for ICI vs controlled therapies		Test for difference	
		Men	Women	Men	Women	*I*^2^, %	*P* value
Overall	32	13 674	7025	0.75 (0.71-0.81)	0.79 (0.72-0.88)	19.02	.25
Cancer type							
NSCLC	14	6728	3951	0.77 (0.72-0.82)	0.76 (0.64-0.89)	46.51	.98
Melanoma	5	1654	1138	0.62 (0.46-0.83)	0.79 (0.67-0.92)	0.06	.22
Gastric or gastroesophageal junction	4	978	395	0.82 (0.64-1.06)	0.99 (0.72-1.37)	0.00	.12
Other	9	4314	1541	0.77 (0.68-0.88)	0.79 (0.65-0.98)	0.00	.81
Line of therapy							
First	13	6626	3182	0.75 (0.67-0.85)	0.72 (0.59-0.89)	47.32	.86
Subsequent	19	7048	3843	0.75 (0.70-0.81)	0.83 (0.75-0.91)	0.00	.09
Agent of immunotherapy^a^							
CTLA-4 inhibitor	8	3551	1653	0.80 (0.69-0.93)	0.86 (0.72-1.03)	8.40	.44
PD-1/PD-L1 inhibitor	25	10 738	5604	0.74 (0.69-0.79)	0.76 (0.67-0.85)	26.52	.56
Intervention therapy^b^							
ICI alone	23	8709	4526	0.75 (0.69-0.81)	0.83 (0.76-0.92)	0.00	.06
ICI combined with non-ICI	10	4965	2499	0.76 (0.67-0.86)	0.69 (0.54-0.88)	44.99	.54

Abbreviations: CTLA-4, cytotoxic T-lymphocyte antigen-4; HR, hazard ratio; ICI, immune checkpoint inhibitors; NSCLC, non–small cell lung cancer; PD-1, programmed cell death receptor 1; PD-L1, programmed cell death receptor ligand 1.

^a^ One trial used both a CTLA-4 inhibitor (ipilimumab) and PD-1 inhibitor (nivolumab) in the intervention group.

^b^ One trial included both IO alone (ipilimumab) and combined therapy (ipilimumab with gp100) in the intervention group.

A total of 34 trials enrolling 21 213 patients reported data on HR for death according to patients’ age. Among the patients, 12 591 were younger than 65 years (59.4%) and 8622 were 65 years or older (40.6%). The statistically significant advantage of immunotherapy over control therapy in OS was found both in younger (<65 years: HR, 0.77; 95% CI, 0.71-0.83) and older (≥65 years: HR, 0.78; 95% CI, 0.72-0.84) patients (eFigure 3 in the Supplement). No significant difference in OS from ICIs was found between the 2 age groups (*P* = .94, *I^2^* = 15.57%) (eFigure 4 in the Supplement; Table 2). Statistically significant heterogeneity was found among the studies for both younger (<65 years: *Q* = 90.33, *P* < .001, *I^2^* = 60.14%) and older (≥65 years: *Q* = 58.49, *P* = .01, *I^2^* = 38.45%) patients. No significant differences in the survival of immunotherapy compared with control therapy were found in subgroup analyses by cancer type, line of therapy, agent of immunotherapy, or intervention therapy (Table 2).

**Table 2.** Differences in Survival Benefit Associated With Immunotherapy in Younger and Older Patients by Subgroups

Variable	Studies, No.	Participants, No.		Pooled HR (95% CI) for ICI vs controlled therapies			Test for difference		
		<65 y	≥65 y	<65 y	≥65 y	*I*^2^, %		*P* value
Overall	34	12 591	8622	0.77 (0.71-0.83)	0.78 (0.72-0.84)	15.57		.94
Cancer type								
NSCLC	14	5732	4661	0.73 (0.65-0.81)	0.80 (0.73-0.87)	0.00		.25
Melanoma	5	1828	897	0.73 (0.57-0.92)	0.70 (0.58-0.83)	22.85		.43
Gastric or gastroesophageal junction	4	746	513	0.89 (0.70-1.12)	0.78 (0.58-1.06)	0.00		.27
Other	11	4285	2551	0.81 (0.70-0.93)	0.80 (0.68-0.95)	42.72		.95
Line of therapy								
First	14	5930	4248	0.73 (0.63-0.84)	0.79 (0.69-0.89)	16.86		.37
Subsequent	20	6661	4374	0.79 (0.73-0.87)	0.77 (0.70-0.84)	13.37		.48
Agent of immunotherapy^a^								
CTLA-4 inhibitor	8	3088	1794	0.76 (0.64-0.91)	0.93 (0.82-1.05)	0.00		.10
PD-1/PD-L1 inhibitor	27	10 027	7086	0.76 (0.70-0.83)	0.75 (0.69-0.81)	26.64		.70
Intervention therapy^b^								
ICI alone	24	7600	5374	0.79 (0.72-0.87)	0.77 (0.71-0.84)	4.48		.44
ICI combined with non-ICI	12	4991	3248	0.73 (0.63-0.84)	0.80 (0.69-0.93)	27.78		.28

Abbreviations: CTLA-4, cytotoxic T-lymphocyte antigen-4; HR, hazard ratio; ICI, immune checkpoint inhibitors; NSCLC, non–small cell lung cancer; PD-1, programmed cell death receptor 1; PD-L1, programmed cell death receptor ligand 1.

^a^ One trial used both a CTLA-4 inhibitor (ipilimumab) and PD-1 inhibitor (nivolumab) in the intervention group.

^b^ Two trials included both IO alone (ipilimumab, atezolizumab) and combined therapy (ipilimumab with gp100, atezolizumab with cobimetinib) in the intervention group.

A total of 30 trials enrolling 19 229 patients reported data on HR for death according to patients’ ECOG PS, which was 0 in 7896 patients (41.1%) and greater than or equal to 1 in 11 333 patients (58.9%). A significant OS advantage of immunotherapy compared with control therapy was observed for both ECOG PS 0 (HR, 0.81; 95% CI, 0.73-0.90) and PS greater than or equal to 1 (HR, 0.79; 95% CI, 0.74-0.84) patients (eFigure 5 in the Supplement). Again, no significant difference in OS advantage obtained with immunotherapy compared with control therapy was found between the patients with different ECOG PS levels (*P* = .74, *I^2^* = 0%) (eFigure 6 in the Supplement; Table 3). Statistically significant heterogeneity was found among both ECOG PS 0 (*Q* = 73.64, *P* < .001, *I^2^* = 57.90%) and PS greater than or equal to 1 (*Q* = 55.69, *P* = .004, *I^2^* = 44.33%) patients. No statistically significant differences were demonstrated in subgroup analyses (Table 3).

**Table 3.** Differences in Survival Benefit Associated With Immunotherapy in Patients With ECOG PS 0 and ECOG PS 1 or Greater by Subgroups

Variable	Studies, No.	Participants, No.		Pooled HR (95% CI) for ICI vs controlled therapies		Test for difference	
		ECOG 0	ECOG≥1	ECOG 0	ECOG≥1	*I*^2^, %	*P* value
Overall	30	7896	11 333	0.81 (0.73-0.90)	0.79 (0.74-0.84)	0.00	.74
Cancer type							
NSCLC	14	3921	6726	0.77 (0.70-0.86)	0.75 (0.68-0.82)	0.00	.80
Melanoma	3	871	448	0.56 (0.28-1.09)	0.72 (0.52-0.99)	41.25	.52
Gastric or gastroesophageal junction	4	507	865	0.91 (0.59-1.40)	0.84 (0.67-1.05)	57.77	.78
Other	9	2597	3294	0.93 (0.79-1.09)	0.86 (0.80-0.94)	0.62	.61
Line of therapy							
First	13	4016	5555	0.80 (0.68-0.94)	0.79 (0.72-0.87)	0.00	.68
Subsequent	17	3880	5778	0.81 (0.72-0.92)	0.78 (0.72-0.85)	11.38	.88
Agent of immunotherapy							
CTLA-4 inhibitor	6	1747	1966	0.95 (0.76-1.20)	0.92 (0.81-1.03)	24.62	.44
PD-1/PD-L1 inhibitor	24	6149	9367	0.78 (0.70-0.86)	0.77 (0.72-0.82)	0.00	.96
Intervention therapy^a^							
ICI alone	21	4792	7224	0.81 (0.71-0.92)	0.79 (0.73-0.86)	14.36	.84
ICI combined with non-ICI	10	3104	4109	0.81 (0.68-0.96)	0.78 (0.70-0.87)	0.00	.34

Abbreviations: CTLA-4, cytotoxic T-lymphocyte antigen-4; ECOG PS, Eastern Cooperative Oncology Group performance status; HR, hazard ratio; ICI, immune checkpoint inhibitors; NSCLC, non–small cell lung cancer; PD-1, programmed cell death receptor 1; PD-L1, programmed cell death receptor ligand 1.

^a^ One trial included both IO alone (atezolizumab) and combined therapy (atezolizumab with cobimetinib) in the intervention group.

## Discussion

To our knowledge, this is the first study to assess the heterogeneity of ICI survival benefit between patients with different ECOG PS and conduct a comprehensive updated analysis of the heterogeneity between patients with different sexes and ages. Our results suggest no evidence of association of sex, age, and ECOG PS with the level of OS benefit from ICIs vs control therapy without ICI.

In terms of the association between sex and the survival benefit of immunotherapy, our results were similar to those of the meta-analysis by Wallis et al^11^ but different from an earlier study performed by Conforti et al,^10^ who reported that men benefited more from immunotherapy. Several reasons may explain these conflicting results. First, the Conforti et al study^10^ included 2 melanoma studies that compared different ICIs in which men appeared to have greater benefit (HR, 0.57 vs 0.69 and 0.65 vs 0.89),^53,54^ in line with the main conclusion of their study. We and Wallis et al^11^ excluded these studies because the focus was to compare ICI immunotherapy with nonimmunotherapy. Second, Conforti et al excluded anti-PD-L1 trials in their study, whereas we and Wallis et al included trials with anti-PD-L1 agents, such as atezolizumab, durvalumab, and avelumab. The large OAK trial^37^ included 467 men and 758 women and found a greater OS advantage among women (HR, 0.79; 95% CI, 0.66-0.93) compared with men (HR, 0.81; 95% CI, 0.65-1.01), but the OAK trial was excluded in the Conforti study.^10^ Third, several large trials that were published after the Conforti et al study showed a greater benefit of immunotherapy in women. The PACIFIC trial^34^ included 500 men and 213 women and showed a stronger OS advantage among women (HR, 0.46; 95% CI, 0.30-0.73) compared with men (HR, 0.78; 95%, CI 0.59-1.03). The KEYNOTE-189 trial^38^ that included 363 men and 253 women demonstrated an OS advantage in women (HR, 0.29; 95% CI, 0.19-0.44) compared with men (HR, 0.70; 95% CI, 0.50-0.99). In KEYNOTE-407,^42^ which included 455 men and 104 women, the OS benefit obtained with immunotherapy compared with control therapy was larger in women (HR, 0.42; 95% CI, 0.22-0.81) than men (HR, 0.69; 95% CI, 0.51-0.94). In CheckMate 214,^41^ which included 615 men and 232 women, the OS benefit was also larger in women (HR, 0.52; 95% CI, 0.34-0.78) than in men (HR, 0.71; 95% CI, 0.55-0.92). In IMpower133,^39^ which included 261 men and 142 women, the benefit of immunotherapy was also larger in women (HR, 0.65; 95% CI, 0.42-1.00) than in men (HR, 0.74; 95% CI, 0.54-1.02). These trials with large sample sizes provide weight significantly in the meta-analysis and likely altered the otherwise positive results shown by Conforti et al.^10^ Compared with Wallis et al,^11^ we included 9 more trials^16,27,35,45,46,48,50,51,52^ that compared ICI immunotherapy with non-ICI control therapy, most of which were new after the Wallis et al meta-analysis.^35,45,46,48,50,51,52^ While some of these larger studies showed more benefit from immunotherapy in men,^16,35,46,48,50^ our updated meta-analysis did not appear to find evidence of a greater benefit from immunotherapy in men or women.

A recent study found that patients older than 60 years responded more efficiently to anti-PD-1 compared with younger patients.^55^ A further animal study reported that older mice had a significantly increased number of CD8^+^ T cells. The fact that CD8^+^ T cells are the primary target cell type of anti-PD1 inhibition might partly explain the better efficacy of ICI in older patients.^55^ A meta-analysis by Wu et al^12^ reported an apparently larger relative benefit from ICI vs control therapy for patients aged 65 years or older than for those younger than 65 years. In contrast, our study did not show a difference of survival benefit associated with immunotherapy in older vs younger patients. The different selection criteria can partly explain the conflicting results. To better assess the association of age with immunotherapy efficacy, we only included trials that compared ICI therapy with control therapy without ICI. However, Wu et al^12^ included 3 trials that compared various immunotherapy regimens, all of which suggested a stronger OS benefit from ICI for older patients compared with younger patients.^53,56,57^ We excluded 2 studies in the analysis by Wu et al because we could not verify the source of subgroup data for the POPLAR trial,^58^ and the other study^59^ was a duplicate of an earlier report of the OAK trial.^37^ Moreover, our updated analysis included a number of more recent large trials that did not show substantial differences of OS benefit from ICI over control therapy in older vs younger patients.^31,33,34,36,38,41,42,45,49,52^ Another meta-analysis by Kasherman et al^13^ also reported no statistically significant age differences in immunotherapy efficacy. However, their study included 4 trials using progression-free survival as the end point and 2 trials using age 70 years as the cutoff, which were all excluded in our analysis. In addition, we included the updated data of the OAK,^37^ KEYNOTE-040,^46^ and KEYNOTE-042^48^ trials, as well as a number of more recent studies.^27,34,35,36,39,44,47,50,51,52^ Our more updated analysis supports the contention that there is no evidence of age difference in immunotherapy survival benefit.

In addition to sex and age, ECOG PS has been reported to potentially affect immune responses.^9^ But to date, little is known whether PS-related alterations in immune response influence antitumor efficacy of ICI. Thus, we also assessed the heterogeneity of immunotherapy survival benefit between better ECOG PS and poorer ECOG PS patients. Again, no statistically significant differences were found in patients with different ECOG PS. Most of the included trials used ECOG PS 0 and ECOG PS 1 to represent better and poorer performance status, respectively, likely owing to stringent inclusion criteria regarding ECOG PS (usually limited to ECOG PS level <2). While our analysis did not demonstrate an association of ECOG PS (0 vs ≥1) with immunotherapy survival benefit, patients with ECOG PS greater than or equal to 2 were underrepresented in the included trials, and these data should be interpreted with caution.

Our findings suggest that there was substantial heterogeneity across studies. The studies assessed the survival benefit of immunotherapy with various designs, including different cancer types, lines of therapy, agents of immunotherapy, and intervention therapies, which may contribute to the between-study heterogeneity. Thus, we performed a stratified analysis to explore whether the association of sex, age, and ECOG PS with survival benefit of immunotherapy was associated with these variables. No significant differences were found in any of the subgroup analyses, although the trial numbers can be small in subset analyses. Taken together, the comparable survival advantage between patients of different sex, age, and ECOG PS may encourage more patients to receive ICI treatment regardless of cancer types, lines of therapy, agents of immunotherapy, and intervention therapies.

### Strengths and Limitations

One of the strengths of this meta-analysis is the comprehensive and up-to-date appropriate study inclusion. We set broad literature search terms and rigorous inclusion criteria to identify the studies that compared immunotherapy with other therapies, and the up-to-date search identified a large number of randomized clinical trials involving more than 23 000 patients, allowing a large meta-analysis. Second, compared with previous studies, we examined the association of cancer immunotherapy survival benefit over control therapy with 3 variables: sex, age, and ECOG PS. Third, we performed a number of subgroup analyses to explore the potential factors that might affect the magnitude of OS benefit from immunotherapy.

Our study also has limitations. First, the analysis relies on published study-level data; lack of individual patient-level data prevents additional analyses, for example, whether different age cutoffs affect the results. Second, this meta-analysis is subject to publication bias given that our analysis was based on published literature. Third, our study cannot fully address the association of age and ECOG PS with ICI OS benefit. For age, the older patients who participate in clinical trials might not represent the whole older population owing to age cutoffs in inclusion criteria in the trials. Moreover, a numeric age cutoff is not sufficient to identify older patients because aging is a variable physiologic process. For ECOG PS, most of the trials used ECOG PS 0 and ECOG PS 1 to dichotomize patients into 2 groups. In reality, ECOG PS 0 to 1 and ECOG PS greater than 1 might be more representative of the 2 groups with different functional status. In addition, we focused on comparing relative OS benefit from ICI over control therapy between groups and did not compare actual survival outcomes between groups when treated with ICI owing to lack of data and did not compare different immunotherapy strategies.

## Conclusions

The findings of this meta-analysis suggest that patients with different sex, age (<65 vs ≥65 years), or ECOG PS (0 vs ≥1) could derive a similar magnitude of survival benefit from ICI immunotherapy compared with control treatments. The use of ICIs in advanced cancer should not be restricted by sex, age, or ECOG PS.

## Notes

Supplement. **eTable.** List of the Studies Included in This Meta-analysis **eFigure 1.** Forest Plots of HRs for OS of Immunotherapy vs Control Therapy in Men and Women **eFigure 2.** Forest Plots of Interaction HRs for OS of Immunotherapy vs Control Therapy by Sex **eFigure 3.** Forest Plots of HRs for OS of Immunotherapy vs Control Therapy in Younger (Age <65) and Older (Age ≥65) Patients **eFigure 4.** Forest Plots of Interaction HRs for OS of Immunotherapy vs Control Therapy by Age **eFigure 5.** Forest Plots of HRs for OS of Immunotherapy vs Control Therapy in ECOG PS 0 and ECOG PS≥1 Patients **eFigure 6.** Forest Plots of Interaction HRs for OS of Immunotherapy vs Control Therapy by ECOG PS
