# Supplementary material for: Association of Sex, Age, and Eastern Cooperative Oncology Group Performance Status With Survival Benefit of Cancer Immunotherapy in Randomized Clinical Trials: A Systematic Review and Meta-analysis
Source: JAMA Netw Open. 2020 Aug 7;3(8):e2012534. doi: 10.1001/jamanetworkopen.2020.12534 (PMC7414387; doi:10.1001/jamanetworkopen.2020.12534)
Supplement: Supplement. — eTable. List of the Studies Included in This Meta-analysis eFigure 1. Forest Plots of HRs for OS of Immunotherapy vs Control Therapy in Men and Women eFigure 2. Forest Plots of Interaction HRs for OS of Immunotherapy vs Control Therapy by Sex eFigure 3. Forest Plots of HRs for OS of Immunotherapy vs Control Therapy in Younger (Age <65) and Older (Age ≥65) Patients eFigure 4. Forest Plots of Interaction HRs for OS of Immunotherapy vs Control Therapy by Age eFigure 5. Forest Plots of HRs for OS of Immunotherapy vs Control Therapy in ECOG PS 0 and ECOG PS≥1 Patients eFigure 6. Forest Plots of Interaction HRs for OS of Immunotherapy vs Control Therapy by ECOG PS [file jamanetwopen-3-e2012534-s001.pdf]

Supplementary Online Content

Yang F, Markovic SN, Molina JR, et al. Association of sex, age, and Eastern Cooperative Oncology Group performance status with survival benefit of cancer immunotherapy in randomized clinical trials: a systematic review and meta-analysis. *JAMA Netw Open*. 2020;3(8):e2012534. doi:10.1001/jamanetworkopen.2020.12534

eTable. List of the Studies Included in This Meta-analysis

eFigure 1. Forest Plots of HRs for OS of Immunotherapy vs Control Therapy in Men and Women

eFigure 2. Forest Plots of Interaction HRs for OS of Immunotherapy vs Control Therapy by Sex

eFigure 3. Forest Plots of HRs for OS of Immunotherapy vs Control Therapy in Younger (Age <65) and Older (Age ≥65) Patients

eFigure 4. Forest Plots of Interaction HRs for OS of Immunotherapy vs Control Therapy by Age

eFigure 5. Forest Plots of HRs for OS of Immunotherapy vs Control Therapy in ECOG PS 0 and ECOG PS ≥1 Patients

eFigure 6. Forest Plots of Interaction HRs for OS of Immunotherapy vs Control Therapy by ECOG PS

This supplementary material has been provided by the authors to give readers additional information about their work.

© 2020 Yang F et al. *JAMA Network Open.*

eTable. List of the studies included in this meta-analysis

Source	Total pt #	Phase	NCT #	Trial name	Cancer type	Line of therapy	Treatment arms	Pt #	OS HR (95%CI)	Sex	Pt #	OS HR (95%CI)	Age	Pt #	OS HR (95% CI)	ECOG PS	Pt #	OS HR (95% CI)
2010-Hodi-N Engl J Med[16]	676	3	NCT00094653		Melanoma	>1	Ipilimumab + gp100 vs gp100	539	0.69 (0.56-0.85)	Male	320	0.66 (0.50-0.87)	<65	385	0.70 (0.54-0.90)			
										Female	219	0.72 (0.52-0.99)	≥65	154	0.69 (0.47-1.01)			
							Ipilimumab vs gp100	273	0.64 (0.49-0.84)	Male	154	0.54 (0.37-0.77)	<65	189	0.65 (0.47-0.90)			
										Female	119	0.81 (0.55-1.20)	≥65	84	0.61 (0.38-0.99)			
2011-Robert-N Engl J Med[17]	502	3	NCT00324155		Melanoma	>1	Ipilimumab + dacarbazine vs Placebo + dacarbazine	502	0.47 (0.30-0.72)	Male	301	0.45 (0.25-0.79)	<65	342	0.44 (0.25-0.74)	0	356	0.54 (0.31-0.91)
										Female	201	0.71 (0.35-1.45)	≥65	160	0.81 (0.36-1.78)	1	146	0.47 (0.21-1.02)
2013-Ribas-J Clin Oncol[18]	655	3	NCT00257205		Melanoma	>1	Tremelimumab vs Standard of care chemotherapy	655		Male	372	0.93 (0.74-1.17)	<65	455	0.88 (0.72-1.08)			
										Female	283	0.81 (0.62-1.06)	≥65	200	0.87 (0.64-1.18)			
2014-Kwon-Lancet Oncol[19]	799	3	NCT00861614	CA184-043	Prostate cancer	>1	Ipilimumab vs Placebo	799	0.85 (0.72-1.00)							0	335	0.72 (0.55-0.94)
																1	464	0.94 (0.76-1.16)
2015-Borghaei-N Engl J Med[20]	582	3	NCT01673867	CheckMate 057	NSCLC	>1	Nivolumab vs Docetaxel	582	0.75 (0.62-0.91)	Male	319	0.73 (0.56-0.96)	<65	339	0.81 (0.62-1.04)	0	179	0.64 (0.44-0.93)
										Female	263	0.78 (0.58-1.04)	≥65 to <75	200	0.63 (0.45-0.89)	1	402	0.80 (0.63-1.00)
2015-Brahmer-N Engl J Med[21]	272	3	NCT01642004		NSCLC	>1	Nivolumab vs Docetaxel	272	0.59 (0.44-0.78)	Male	208	0.57 (0.41-0.78)	<65	152	0.52 (0.35-0.75)	0	64	0.48 (0.24-0.99)
										Female	64	0.67 (0.36-1.25)	≥65 to <75	91	0.56 (0.34-0.91)	1	206	0.54 (0.39-0.74)
2015-Motzer-N Engl J Med[22]	821	3	NCT01668784	CheckMate 025	Renal cell carcinoma	>1	Nivolumab vs Exerolimus	821	0.76 (0.62-0.92)	Male	619	0.73 (0.58-0.92)	<65	497	0.78 (0.60-1.01)			
										Female	202	0.84 (0.57-1.24)	≥65 to <75	250	0.64 (0.45-0.91)			
2015-Robert-N Engl J Med[23]	418	3	NCT01721772	CheckMate 066	Melanoma	1	Nivolumab vs Dacarbazine	418	0.42 (0.30-0.59)	Male	246	0.34 (0.22-0.54)	<65	200	0.52 (0.32-0.85)	0	269	0.32 (0.20-0.53)
										Female	172	0.56 (0.33-0.95)	≥65 to <75	151	0.44 (0.24-0.81)	1	144	0.64 (0.40-1.04)
2016-Ferris-N Engl J Med[24]	361	3	NCT02105636	CheckMate 141	Head and neck carcinoma	>1	Nivolumab vs Standard therapy	361	0.69 (0.53-0.91)	Male	300	0.65 (0.48-0.88)	<65	248	0.64 (0.45-0.89)	0	72	0.60 (0.30-1.23)

© 2020 Yang F et al. *JAMA Network Open.*

										Female	61	0.93 (0.47-1.85)	≥65 to <75	95	0.93 (0.56-1.54)	≥1	287	0.71 (0.53-0.96)
2016-Herbst-Lancet[25]	1033	2/3	NCT01905657	KEYNOTE-010	NSCLC	>1	Pembrolizumab vs Docetaxel	1033	0.67 (0.56-0.80)	Male	634	0.65 (0.52-0.81)	<65	604	0.63 (0.50-0.79)	0	348	0.73 (0.52-1.02)
										Female	399	0.69 (0.51-0.94)	≥65	429	0.76 (0.57-1.02)	1	678	0.63 (0.51-0.78)
2016-Reck-J Clin Oncol[26]	954	3	NCT01450761		SCLC	1	Ipilimumab + etoposide + platinum vs Placebo + etoposide + platinum	954	0.94 (0.81-1.09)	Male	643	1.07 (0.89-1.28)	<65	576	1.08 (0.90-1.31)	0	284	1.28 (0.98-1.69)
										Female	311	1.06 (0.81-1.37)	≥65 to <75	306	1.14 (0.87-1.49)	1	668	0.99 (0.83-1.18)
2017-Bang-Clin Cancer Res[27]	114	2	NCT01585987		Gastric or gastro-oesophageal junction cancer	>1	Ipilimumab vs Best supportive care	114		Male	77	0.95 (0.66-1.34)	<65		0.91 (0.57-1.48)	0	56	1.22 (0.70-2.12)
										Female	37	0.93 (0.47-1.83)	≥65		0.90 (0.50-1.59)	1	58	0.69 (0.41-1.16)
2017-Beer-J Clin Oncol[28]	602	3	NCT01057810		Prostate cancer	1	Ipilimumab vs Placebo	602	1.11 (0.88-1.39)							0	457	1.09 (0.85-1.40)
																1	145	1.08 (0.72-1.62)
2017-Bellmunt-N Engl J Med[29]	542	3	NCT02256436	KEYNOTE-045	Urothelial carcinoma	>1	Pembrolizumab vs Chemotherapy	542	0.73 (0.59-0.91)	Male	402	0.73 (0.56-0.94)	<65	230	0.75 (0.53-1.05)			
										Female	140	0.78 (0.49-1.24)	≥65	312	0.76 (0.56-1.02)			
2017-Carbone-N Engl J Med[30]	541	3	NCT02041533		NSCLC	1	Nivolumab vs Chemotherapy	541	1.08 (0.87-1.34)	Male	332	0.97 (0.74-1.26)	<65	281	1.13 (0.83-1.54)	0	178	1.11 (0.74-1.66)
										Female	209	1.15 (0.79-1.66)	≥65	260	1.04 (0.77-1.41)	≥1	362	1.02 (0.79-1.32)
2017-Govindan-J Clin Oncol[31]	749	3	NCT01285609		NSCLC	1	Ipilimumab + chemotherapy vs Placebo + chemotherapy	749	0.91 (0.77-1.07)	Male	635	0.85 (0.71-1.02)	<65	380	0.82 (0.64-1.04)	0	259	0.99 (0.73-1.33)
										Female	114	1.33 (0.84-2.11)	≥65 to <75	298	1.06 (0.81-1.37)	1	485	0.86 (0.70-1.05)
2017-Kang-Lancet[32]	493	3	NCT02267343	ONO-4538-12, ATTRACTION-2	Gastric or gastro-oesophageal junction cancer	>1	Nivolumab vs Placebo	493	0.65 (0.53-0.80)	Male	348	0.59 (0.46-0.75)	<65	284	0.76 (0.58-1.00)	0	143	0.59 (0.40-0.87)
										Female	145	0.83 (0.56-1.23)	≥65	209	0.53 (0.38-0.74)	1	350	0.68 (0.53-0.87)
2017-Maio-Lancet Oncol[33]	571	2b	NCT01843374	DETERMINE	Mesothelioma	>1	Tremelimumab vs Placebo	571	0.92 (0.76-1.12)	Male	434	0.91 (0.73-1.13)	<65	237	0.87 (0.64-1.20)			
										Female	137	1.12 (0.72-1.75)	≥65	334	0.99 (0.77-1.26)			
2018-Antonia-N Engl J Med[34]	713	3	NCT02125461	PACIFIC	NSCLC	>1	Durvalumab vs Placebo	713	0.68 (0.54-0.86)	Male	500	0.78 (0.59-1.03)	<65	391	0.62 (0.44-0.86)	0	348	0.82 (0.57-1.16)

© 2020 Yang F et al. *JAMA Network Open.*

										Female	213	0.46 (0.30-0.73)	≥65	322	0.76 (0.55-1.06)	1	365	0.58 (0.42-0.79)
2018-Bang-Ann Oncol[35]	371	3	NCT02625623	JAVELIN Gastric 300	Gastric or gastro-oesophageal junction cancer	>1	Avelumab vs Chemotherapy	371	1.1 (0.90-1.40)	Male	267	0.99 (0.75-1.32)	<65	230	1.22 (0.89-1.65)	0	128	1.48 (0.97-2.27)
										Female	104	1.54 (0.99-2.38)	≥65	141	0.95 (0.65-1.38)	1	243	1.01 (0.76-1.35)
2018-Barlesi-Lancet Oncol[36]	529	3	NCT02395172	JAVELIN Lung 200	NSCLC	>1	Avelumab vs Docetaxel	529	0.90 (0.73-1.12)	Male	367	0.83 (0.64-1.08)	<65	279	0.84 (0.63-1.13)	0	187	0.73 (0.50-1.08)
										Female	162	1.08 (0.74-1.59)	≥65	250	0.98 (0.71-1.34)	1	342	0.99 (0.77-1.28)
2018-Fehrenbacher-J Thorac Oncol[37]	1225	3	NCT02008227	OAK	NSCLC	>1	Atezolizumab vs Docetaxel	1225	0.80 (0.70-0.92)	Male	467	0.81 (0.65-1.01)	<65	661	0.84 (0.70-1.01)	0	455	0.80 (0.63-1.02)
										Female	758	0.79 (0.66-0.93)	≥65	564	0.75 (0.61-0.91)	1	770	0.77 (0.65-0.90)
2018-Gandhi-N Engl J Med[38]	616	3	NCT02578680	KEYNOTE-189	NSCLC	1	Pembrolizumab + chemotherapy vs Placebo + chemotherapy	616	0.49 (0.38-0.64)	Male	363	0.70 (0.50-0.99)	<65	312	0.43 (0.31-0.61)	0	266	0.44 (0.28-0.71)
										Female	253	0.29 (0.19-0.44)	≥65	304	0.64 (0.43-0.95)	1	346	0.53 (0.39-0.73)
2018-Horn-N Engl J Med[39]	403	3	NCT02763579	IMpower133	SCLC	1	Atezolizumab + chemotherapy vs Placebo + chemotherapy	403	0.70 (0.54-0.91)	Male	261	0.74 (0.54-1.02)	<65	217	0.92 (0.64-1.32)	0	140	0.79 (0.49-1.27)
										Female	142	0.65 (0.42-1.00)	≥65	186	0.53 (0.36-0.77)	1	263	0.68 (0.50-0.93)
2018-Larkin-J Clin Oncol[40]	405	3	NCT01721746	CheckMate 037	Melanoma	>1	Nivolumab vs Chemotherapy	405	0.92 (0.71-1.18)	Male	261	0.85 (0.62-1.17)	<65	257	1.17 (0.84-1.63)	0	246	0.95 (0.67-1.34)
										Female	144	1.07 (0.69-1.65)	≥65	148	0.62 (0.41-0.94)	1	158	0.89 (0.60-1.31)
2018-Motzer-N Engl J Med[41]	847	3	NCT02231749	CheckMate 214	Renal cell carcinoma	1	Nivolumab + ipilimumab vs Sunitinib	847	0.66 (0.53-0.82)	Male	615	0.71 (0.55-0.92)	<65	524	0.53 (0.40-0.71)			
										Female	232	0.52 (0.34-0.78)	≥65 to <75	258	0.86 (0.58-1.27)			
2018-Paz-Ares-N Engl J Med[42]	559	3	NCT02775435	KEYNOTE-407	NSCLC	1	Pembrolizumab + chemotherapy vs Placebo + chemotherapy	559	0.64 (0.49-0.85)	Male	455	0.69 (0.51-0.94)	<65	254	0.52 (0.34-0.80)	0	163	0.54 (0.29-0.98)
										Female	104	0.42 (0.22-0.81)	≥65	309	0.74 (0.51-1.07)	1	396	0.66 (0.48-0.90)
2018-Powles-Lancet[43]	931	3	NCT02302807	IMvigor211	Urothelial carcinoma	>1	Atezolizumab vs Chemotherapy	931	0.84 (0.72-0.97)							0	425	0.84 (0.66-1.06)
																1	506	0.87 (0.71-1.05)

© 2020 Yang F et al. *JAMA Network Open.*

2018-Schmid-N Engl J Med[44]	902	3	NCT02425891	IMpassion130	TNBC	1	Atezolizumab + nab-paclitaxel vs Placebo + nab-paclitaxel	902	0.81 (0.70-0.93)				41-64	569	0.84 (0.70-1.01)	0	526	0.78 (0.64-0.94)
													>65	219	0.69 (0.51-0.94)	1	372	0.82 (0.66-1.03)
2018-Shitara-Lancet[45]	395	3	NCT02370498	KEYNOTE-061	Gastric or gastro-oesophageal junction cancer	>1	Pembrolizumab vs Paclitaxel	395	0.82 (0.66-1.03)	Male	286	0.87 (0.67-1.14)	≤65	232	0.77 (0.58-1.02)	0	180	0.69 (0.49-0.97)
										Female	109	0.81 (0.52-1.26)	>65	163	0.90 (0.63-1.29)	1	214	0.98 (0.73-1.32)
2019-Cohen-Lancet[46]	495	3	NCT02252042	KEYNOTE-040	Head and neck carcinoma	>1	Pembrolizumab vs Standard of care	495	0.80 (0.65-0.98)	Male	412	0.77 (0.62-0.96)	<65	332	0.94 (0.73-1.20)	0	138	0.87 (0.57-1.32)
										Female	83	0.94 (0.54-1.63)	≥65 to <75	132	0.57 (0.37-0.87)	1	356	0.78 (0.62-0.98)
2019-Eng-Lancet Oncol[47]	363	3	NCT02788279	IMblaze370	Colorectal cancer	>1	Atezolizumab + cobimetinib vs Regorafenib	273	1.01 (0.74-1.38)				<65	187	0.84 (0.58-1.23)	0	133	1.13 (0.71-1.82)
													≥65	86	1.50 (0.85-2.64)	1	140	0.85 (0.56-1.30)
							Atezolizumab vs Regorafenib	180	1.18 (0.83-1.69)				<65	130	1.20 (0.80-1.81)	0	87	1.65 (0.96-2.81)
													≥65	50	0.99 (0.47-2.07)	1	93	0.85 (0.52-1.37)
2019-Mok-Lancet[48]	1274	3	NCT02220894	KEYNOTE-042	NSCLC	1	Pembrolizumab vs Chemotherapy (PD-L1 TPS ≥1%)	1274	0.81 (0.71-0.93)	Male	902	0.80 (0.68-0.94)	<65	707	0.81 (0.67-0.98)	0	390	0.77 (0.58-1.05)
										Female	372	0.89 (0.68-1.17)	≥65	567	0.82 (0.66-1.01)	1	884	0.83 (0.71-0.98)
2019-Reck-J Clin Oncol[49]	305	3	NCT02142738	KEYNOTE-024	NSCLC	1	Pembrolizumab vs Platinum-based chemotherapy	305	0.63 (0.47-0.86)	Male	187	0.54 (0.36-0.79)	<65	141	0.60 (0.38-0.96)	0	107	0.78 (0.44-1.37)
										Female	118	0.95 (0.56-1.62)	≥65	164	0.64 (0.42-0.98)	1	197	0.56 (0.39-0.81)
2019-Reck-Lancet Respir Med[50]	1202	3	NCT02366143	IMpower150	NSCLC	1	ABCP vs BCP	800	0.76 (0.63-0.93)	Male	479	0.73 (0.57-0.93)	<65	441	0.78 (0.60-1.00)	0	338	0.75 (0.53-1.07)
										Female	321	0.82 (0.61-1.12)	65-74	281	0.69 (0.49-0.96)	1	456	0.75 (0.59-0.94)
							ACP vs BCP	802	0.85 (0.71-1.03)	Male	480	0.82 (0.64-1.04)	<65	449	0.76 (0.59-0.98)	0	359	0.85 (0.61-1.18)
										Female	322	0.88 (0.65-1.19)	65-74	284	0.97 (0.71-1.32)	1	440	0.84 (0.67-1.06)
2019-Rini-N Engl J Med[51]	861	3	NCT02853331	KEYNOTE-426	Renal cell carcinoma	1	Pembrolizumab + axitinib vs Sunitinib	861	0.53 (0.38-0.74)	Male	628	0.54 (0.37-0.80)	<65	538	0.47 (0.30-0.73)			
										Female	233	0.45 (0.25-0.83)	≥65	323	0.59 (0.36-0.97)			

© 2020 Yang F et al. *JAMA Network Open.*

2019-West-Lancet Oncol[52]	679	3	NCT02367781	IMpower130	NSCLC	1	Atezolizumab + carboplatin + nab-paclitaxel vs Chemotherapy	679	0.80 (0.65-0.99)	Male	400	0.87 (0.66-1.15)	<65	341	0.79 (0.58-1.08)	0	280	0.85 (0.59-1.22)
										Female	279	0.66 (0.46-0.93)	≥65	338	0.78 (0.58-1.05)	1	397	0.77 (0.58-1.00)

Pt: patient; OS: overall survival; HR: hazard ratio; CI: confidence interval; ECOG PS: Eastern Cooperative Oncology Group Performance Status; ABCP: atezolizumab + bevacizumab + carboplatin + paclitaxel; BCP: bevacizumab + carboplatin + paclitaxel; ACP: atezolizumab + carboplatin + paclitaxel; NSCLC: non-small cell lung cancer; SCLC: small cell lung cancer; TNBC: triple-negative breast cancer.

© 2020 Yang F et al. *JAMA Network Open.*

eFigure 1. Forest plots of HRs for OS of immunotherapy vs control therapy in (A) men and (B) women.

IO: immunotherapy; OS: overall survival; HRs: hazard ratios; CI: confidence interval; ABCP: atezolizumab + bevacizumab + carboplatin + paclitaxel; BCP: bevacizumab + carboplatin + paclitaxel; ACP: atezolizumab + carboplatin + paclitaxel.

A

Study name	Comparison	Hazard ratio	Lower limit	Upper limit	p-Value
Hodi et al (2010)	Ipilimumab+gp100 vs gp100	0.66	0.50	0.87	0.003
Hodi et al (2010)	Ipilimumab vs gp100	0.54	0.37	0.78	0.001
Robert et al (2011)	Ipilimumab+dacarbazine vs Placebo+dacarbazine	0.45	0.25	0.80	0.007
Ribas et al (2013)	Tremelimumab vs Standard of care chemotherapy	0.93	0.74	1.17	0.535
Borghaei et al (2015)	Nivolumab vs Docetaxel	0.73	0.56	0.96	0.022
Brahmer et al (2015)	Nivolumab vs Docetaxel	0.57	0.41	0.79	0.001
Motzer et al (2015)	Nivolumab vs Exerolimus	0.73	0.58	0.92	0.007
Robert et al (2015)	Nivolumab vs Dacarbazine	0.34	0.22	0.53	0.000
Ferris et al (2016)	Nivolumab vs Standard therapy	0.65	0.48	0.88	0.005
Herbst et al (2016)	Pembrolizumab vs Docetaxel	0.65	0.52	0.81	0.000
Reck et al (2016)	Ipilimumab+etoposide+platinum vs Placebo+etoposide+platinum	1.07	0.89	1.28	0.465
Bang et al (2017)	Ipilimumab vs Best supportive care	0.95	0.67	1.35	0.776
Bellmunt et al (2017)	Pembrolizumab vs Chemotherapy	0.73	0.56	0.95	0.017
Carbone et al (2017)	Nivolumab vs Chemotherapy	0.97	0.74	1.27	0.822
Govindan et al (2017)	Ipilimumab+chemotherapy vs Placebo+chemotherapy	0.85	0.71	1.02	0.079
Kang et al (2017)	Nivolumab vs Placebo	0.59	0.46	0.75	0.000
Maio et al (2017)	Tremelimumab vs Placebo	0.91	0.73	1.13	0.397
Antonia et al (2018)	Durvalumab vs Placebo	0.78	0.59	1.03	0.080
Bang et al (2018)	Avelumab vs Chemotherapy	0.99	0.75	1.31	0.944
Barlesi et al (2018)	Avelumab vs Docetaxel	0.83	0.64	1.08	0.163
Fehrenbacher et al (2018)	Atezolizumab vs Docetaxel	0.81	0.65	1.01	0.061
Gandhi et al (2018)	Pembrolizumab+chemotherapy vs Placebo+chemotherapy	0.70	0.50	0.98	0.041
Horn et al (2018)	Atezolizumab+chemotherapy vs Placebo+chemotherapy	0.74	0.54	1.02	0.063
Larkin et al (2018)	Nivolumab vs Chemotherapy	0.85	0.62	1.17	0.316
Motzer et al (2018)	Nivolumab+ipilimumab vs Sunitinib	0.71	0.55	0.92	0.009
Paz-Ares et al (2018)	Pembrolizumab+chemotherapy vs Placebo+chemotherapy	0.69	0.51	0.94	0.017
Shitara et al (2018)	Pembrolizumab vs Paclitaxel	0.87	0.67	1.13	0.304
Cohen et al (2019)	Pembrolizumab vs Standard of care	0.77	0.62	0.96	0.019
Mok et al (2019)	Pembrolizumab vs Chemotherapy	0.80	0.68	0.94	0.007
Reck et al (2019)	Pembrolizumab vs Platinum-based chemotherapy	0.54	0.36	0.80	0.002
Reck et al (2019)	ABCP vs BCP	0.73	0.57	0.93	0.012
Reck et al (2019)	ACP vs BCP	0.82	0.64	1.05	0.109
Rini et al (2019)	Pembrolizumab+axitinib vs Sunitinib	0.54	0.37	0.79	0.002
West et al (2019)	Atezolizumab+carboplatin+nab-paclitaxel vs Chemotherapy	0.87	0.66	1.15	0.326
		0.75	0.71	0.81	0.000

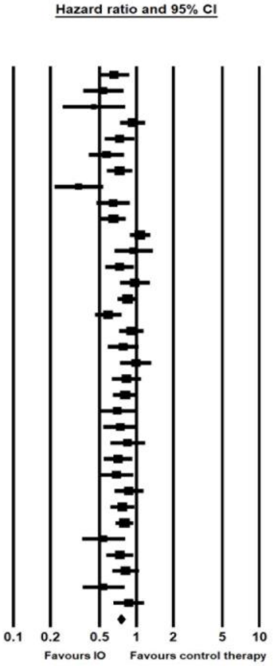

Overall survival for men

B

Study name	Comparison	Hazard ratio	Lower limit	Upper limit	p-Value
Hodi et al (2010)	Ipilimumab+gp100 vs gp100	0.72	0.52	0.99	0.046
Hodi et al (2010)	Ipilimumab vs gp100	0.81	0.55	1.20	0.290
Robert et al (2011)	Ipilimumab+dacarbazine vs Placebo+dacarbazine	0.71	0.35	1.45	0.345
Ribas et al (2013)	Tremelimumab vs Standard of care chemotherapy	0.81	0.62	1.06	0.124
Borghaei et al (2015)	Nivolumab vs Docetaxel	0.78	0.58	1.04	0.095
Brahmer et al (2015)	Nivolumab vs Docetaxel	0.67	0.36	1.25	0.207
Motzer et al (2015)	Nivolumab vs Exerolimus	0.84	0.57	1.24	0.379
Robert et al (2015)	Nivolumab vs Dacarbazine	0.56	0.33	0.95	0.032
Ferris et al (2016)	Nivolumab vs Standard therapy	0.93	0.47	1.85	0.836
Herbst et al (2016)	Pembrolizumab vs Docetaxel	0.69	0.51	0.94	0.017
Reck et al (2016)	Ipilimumab+etoposide+platinum vs Placebo+etoposide+platinum	1.06	0.82	1.38	0.664
Bang et al (2017)	Ipilimumab vs Best supportive care	0.93	0.47	1.84	0.834
Bellmunt et al (2017)	Pembrolizumab vs Chemotherapy	0.78	0.49	1.24	0.294
Carbone et al (2017)	Nivolumab vs Chemotherapy	1.15	0.79	1.67	0.461
Govindan et al (2017)	Ipilimumab+chemotherapy vs Placebo+chemotherapy	1.33	0.84	2.11	0.225
Kang et al (2017)	Nivolumab vs Placebo	0.83	0.56	1.23	0.353
Maio et al (2017)	Tremelimumab vs Placebo	1.12	0.72	1.75	0.617
Antonia et al (2018)	Durvalumab vs Placebo	0.46	0.29	0.72	0.001
Bang et al (2018)	Avelumab vs Chemotherapy	1.54	0.99	2.39	0.054
Barlesi et al (2018)	Avelumab vs Docetaxel	1.08	0.74	1.58	0.693
Fehrenbacher et al (2018)	Atezolizumab vs Docetaxel	0.79	0.67	0.94	0.007
Gandhi et al (2018)	Pembrolizumab+chemotherapy vs Placebo+chemotherapy	0.29	0.19	0.44	0.000
Horn et al (2018)	Atezolizumab+chemotherapy vs Placebo+chemotherapy	0.65	0.42	1.00	0.052
Larkin et al (2018)	Nivolumab vs Chemotherapy	1.07	0.69	1.65	0.761
Motzer et al (2018)	Nivolumab+ipilimumab vs Sunitinib	0.52	0.34	0.79	0.002
Paz-Ares et al (2018)	Pembrolizumab+chemotherapy vs Placebo+chemotherapy	0.42	0.22	0.81	0.009
Shitara et al (2018)	Pembrolizumab vs Paclitaxel	0.81	0.52	1.26	0.351
Cohen et al (2019)	Pembrolizumab vs Standard of care	0.94	0.54	1.63	0.826
Mok et al (2019)	Pembrolizumab vs Chemotherapy	0.89	0.68	1.17	0.400
Reck et al (2019)	Pembrolizumab vs Platinum-based chemotherapy	0.95	0.56	1.62	0.850
Reck et al (2019)	ABCP vs BCP	0.82	0.61	1.11	0.200
Reck et al (2019)	ACP vs BCP	0.88	0.65	1.19	0.407
Rini et al (2019)	Pembrolizumab+axitinib vs Sunitinib	0.45	0.25	0.82	0.009
West et al (2019)	Atezolizumab+carboplatin+nab-paclitaxel vs Chemotherapy	0.66	0.46	0.94	0.021
		0.79	0.72	0.88	0.000

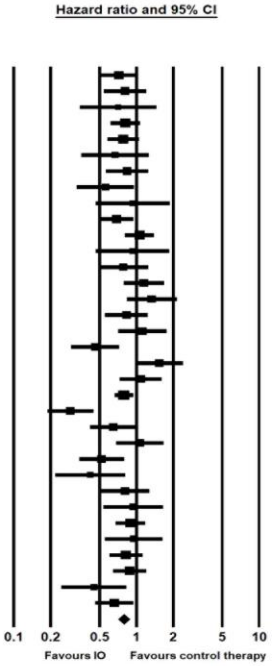

Overall survival for women

© 2020 Yang F et al. *JAMA Network Open.*

eFigure 2. Forest plots of interaction HRs for OS of immunotherapy vs control therapy by sex.

Study-specific interaction HRs for each trial are represented by the squares, where the size of the square represents the weight of the trial in the meta-analysis and the horizontal line crossing the square represents the 95% CI. IO: immunotherapy; OS: overall survival; HRs: hazard ratios; CI: confidence interval; ABCP: atezolizumab + bevacizumab + carboplatin + paclitaxel; BCP: bevacizumab + carboplatin + paclitaxel; ACP: atezolizumab + carboplatin + paclitaxel.

Study name	Comparison	Statistics for each study			
		Hazard ratio	Lower limit	Upper limit	p-Value
Hodi et al (2010)	Ipilimumab+gp100 vs gp100	0.92	0.60	1.41	0.700
Hodi et al (2010)	Ipilimumab vs gp100	0.67	0.39	1.15	0.143
Robert et al (2011)	Ipilimumab+dacarbazine vs Placebo+dacarbazine	0.63	0.25	1.58	0.326
Ribas et al (2013)	Tremelimumab vs Standard of care chemotherapy	1.15	0.81	1.63	0.433
Borghaei et al (2015)	Nivolumab vs Docetaxel	0.94	0.63	1.40	0.759
Brahmer et al (2015)	Nivolumab vs Docetaxel	0.85	0.42	1.72	0.650
Motzer et al (2015)	Nivolumab vs Exerolimus	0.87	0.55	1.37	0.550
Robert et al (2015)	Nivolumab vs Dacarbazine	0.61	0.30	1.23	0.165
Ferris et al (2016)	Nivolumab vs Standard therapy	0.70	0.33	1.48	0.352
Herbst et al (2016)	Pembrolizumab vs Docetaxel	0.94	0.65	1.36	0.745
Reck et al (2016)	Ipilimumab+etoposide+platinum vs Placebo+etoposide+platinum	1.01	0.73	1.39	0.952
Bang et al (2017)	Ipilimumab vs Best supportive care	1.02	0.47	2.21	0.960
Bellmunt et al (2017)	Pembrolizumab vs Chemotherapy	0.94	0.55	1.60	0.819
Carbone et al (2017)	Nivolumab vs Chemotherapy	0.84	0.53	1.33	0.458
Govindan et al (2017)	Ipilimumab+chemotherapy vs Placebo+chemotherapy	0.64	0.39	1.05	0.077
Kang et al (2017)	Nivolumab vs Placebo	0.71	0.45	1.13	0.145
Maio et al (2017)	Tremelimumab vs Placebo	0.81	0.50	1.32	0.398
Antonia et al (2018)	Durvalumab vs Placebo	1.70	1.00	2.88	0.049
Bang et al (2018)	Avelumab vs Chemotherapy	0.64	0.38	1.08	0.094
Barlesi et al (2018)	Avelumab vs Docetaxel	0.77	0.48	1.23	0.272
Fehrenbacher et al (2018)	Atezolizumab vs Docetaxel	1.03	0.78	1.36	0.835
Gandhi et al (2018)	Pembrolizumab+chemotherapy vs Placebo+chemotherapy	2.41	1.40	4.15	0.002
Horn et al (2018)	Atezolizumab+chemotherapy vs Placebo+chemotherapy	1.14	0.66	1.96	0.635
Larkin et al (2018)	Nivolumab vs Chemotherapy	0.79	0.46	1.36	0.394
Motzer et al (2018)	Nivolumab+ipilimumab vs Sunitinib	1.37	0.84	2.23	0.206
Paz-Ares et al (2018)	Pembrolizumab+chemotherapy vs Placebo+chemotherapy	1.64	0.80	3.37	0.178
Shitara et al (2018)	Pembrolizumab vs Paclitaxel	1.07	0.64	1.79	0.798
Cohen et al (2019)	Pembrolizumab vs Standard of care	0.82	0.45	1.49	0.513
Mok et al (2019)	Pembrolizumab vs Chemotherapy	0.90	0.66	1.23	0.507
Reck et al (2019)	Pembrolizumab vs Platinum-based chemotherapy	0.57	0.29	1.11	0.098
Reck et al (2019)	ABCP vs BCP	0.89	0.60	1.32	0.562
Reck et al (2019)	ACP vs BCP	0.93	0.63	1.37	0.714
Rini et al (2019)	Pembrolizumab+axitinib vs Sunitinib	1.20	0.59	2.45	0.616
West et al (2019)	Atezolizumab+carboplatin+nab-paclitaxel vs Chemotherapy	1.32	0.84	2.07	0.225
		0.95	0.87	1.04	0.251

Hazard ratio and 95% CI

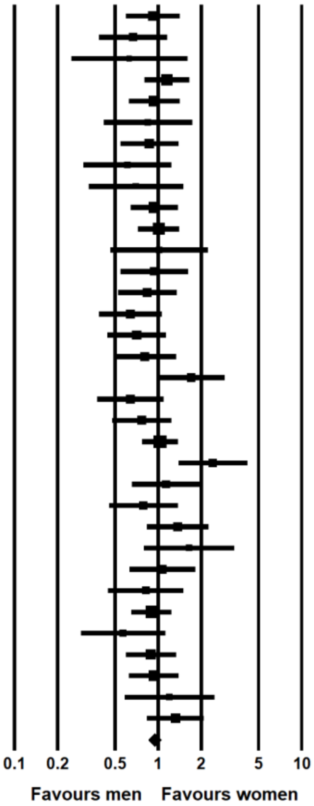

Overall survival for men vs women

© 2020 Yang F et al. *JAMA Network Open.*

eFigure 3. Forest plots of HRs for OS of immunotherapy vs control therapy in (A) younger (age <65) and (B) older (age ≥65) patients

IO: immunotherapy; OS: overall survival; HRs: hazard ratios; CI: confidence interval; ABCP: atezolizumab + bevacizumab + carboplatin + paclitaxel; BCP: bevacizumab + carboplatin + paclitaxel; ACP: atezolizumab + carboplatin + paclitaxel.

A

Study name	Comparison	Hazard ratio	Lower limit	Upper limit	p-Value
Hodi et al (2010)	Ipilimumab+gp100 vs gp100	0.70	0.54	0.90	0.006
Hodi et al (2010)	Ipilimumab vs gp100	0.65	0.47	0.90	0.009
Robert et al (2011)	Ipilimumab+dacarbazine vs Placebo+dacarbazine	0.44	0.26	0.76	0.003
Ribas et al (2013)	Tremelimumab vs Standard of care chemotherapy	0.88	0.72	1.08	0.217
Borghaei et al (2015)	Nivolumab vs Docetaxel	0.81	0.63	1.05	0.110
Brahmer et al (2015)	Nivolumab vs Docetaxel	0.52	0.36	0.76	0.001
Motzer et al (2015)	Nivolumab vs Exerolimus	0.78	0.60	1.01	0.061
Robert et al (2015)	Nivolumab vs Dacarbazine	0.52	0.32	0.85	0.009
Ferris et al (2016)	Nivolumab vs Standard therapy	0.64	0.46	0.90	0.010
Herbst et al (2016)	Pembrolizumab vs Docetaxel	0.63	0.50	0.79	0.000
Reck et al (2016)	Ipilimumab+etoposide+platinum vs Placebo+etoposide+platinum	1.08	0.90	1.30	0.422
Bang et al (2017)	Ipilimumab vs Best supportive care	0.91	0.56	1.47	0.698
Bellmunt et al (2017)	Pembrolizumab vs Chemotherapy	0.75	0.53	1.06	0.099
Carbone et al (2017)	Nivolumab vs Chemotherapy	1.13	0.83	1.54	0.438
Govindan et al (2017)	Ipilimumab+chemotherapy vs Placebo+chemotherapy	0.82	0.64	1.05	0.109
Kang et al (2017)	Nivolumab vs Placebo	0.76	0.58	1.00	0.048
Maio et al (2017)	Tremelimumab vs Placebo	0.87	0.64	1.19	0.385
Antonia et al (2018)	Durvalumab vs Placebo	0.62	0.44	0.87	0.005
Bang et al (2018)	Avelumab vs Chemotherapy	1.22	0.90	1.66	0.207
Barlesi et al (2018)	Avelumab vs Docetaxel	0.84	0.63	1.12	0.242
Fehrenbacher et al (2018)	Atezolizumab vs Docetaxel	0.84	0.70	1.01	0.062
Gandhi et al (2018)	Pembrolizumab+chemotherapy vs Placebo+chemotherapy	0.43	0.31	0.60	0.000
Horn et al (2018)	Atezolizumab+chemotherapy vs Placebo+chemotherapy	0.92	0.64	1.32	0.652
Larkin et al (2018)	Nivolumab vs Chemotherapy	1.17	0.84	1.63	0.353
Motzer et al (2018)	Nivolumab+ipilimumab vs Sunitinib	0.53	0.40	0.71	0.000
Paz-Ares et al (2018)	Pembrolizumab+chemotherapy vs Placebo+chemotherapy	0.52	0.34	0.80	0.003
Schmid et al (2018)	Atezolizumab+nab-paclitaxel vs Placebo+nab-paclitaxel	0.84	0.70	1.01	0.062
Shitara et al (2018)	Pembrolizumab vs Paclitaxel	0.77	0.58	1.02	0.070
Cohen et al (2019)	Pembrolizumab vs Standard of care	0.94	0.73	1.21	0.626
Eng et al (2019)	Atezolizumab+cobimetinib vs Regorafenib	0.84	0.58	1.22	0.363
Eng et al (2019)	Atezolizumab vs Regorafenib	1.20	0.80	1.80	0.381
Mok et al (2019)	Pembrolizumab vs Chemotherapy	0.81	0.67	0.98	0.030
Reck et al (2019)	Pembrolizumab vs Platinum-based chemotherapy	0.60	0.38	0.95	0.031
Reck et al (2019)	ABCP vs BCP	0.78	0.60	1.01	0.057
Reck et al (2019)	ACP vs BCP	0.76	0.59	0.98	0.034
Rini et al (2019)	Pembrolizumab+axitinib vs Sunitinib	0.47	0.30	0.73	0.001
West et al (2019)	Atezolizumab+carboplatin+nab-paclitaxel vs Chemotherapy	0.79	0.58	1.08	0.137
		0.77	0.71	0.83	0.000

Hazard ratio and 95% CI

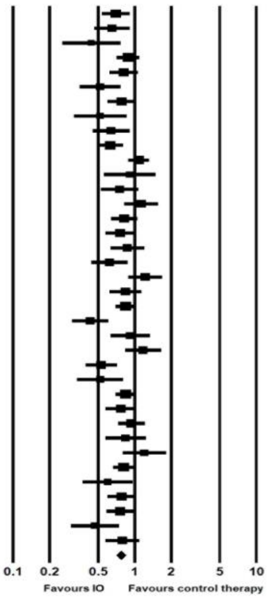

Overall survival for <65 years old

B

Study name	Comparison	Hazard ratio	Lower limit	Upper limit	p-Value
Hodi et al (2010)	Ipilimumab+gp100 vs gp100	0.69	0.47	1.01	0.057
Hodi et al (2010)	Ipilimumab vs gp100	0.61	0.38	0.98	0.043
Robert et al (2011)	Ipilimumab+dacarbazine vs Placebo+dacarbazine	0.81	0.36	1.80	0.605
Ribas et al (2013)	Tremelimumab vs Standard of care chemotherapy	0.87	0.64	1.18	0.372
Borghaei et al (2015)	Nivolumab vs Docetaxel	0.63	0.45	0.89	0.008
Brahmer et al (2015)	Nivolumab vs Docetaxel	0.56	0.34	0.92	0.021
Motzer et al (2015)	Nivolumab vs Exerolimus	0.64	0.45	0.91	0.013
Robert et al (2015)	Nivolumab vs Dacarbazine	0.44	0.24	0.81	0.008
Ferris et al (2016)	Nivolumab vs Standard therapy	0.93	0.56	1.54	0.779
Herbst et al (2016)	Pembrolizumab vs Docetaxel	0.76	0.57	1.02	0.065
Reck et al (2016)	Ipilimumab+etoposide+platinum vs Placebo+etoposide+platinum	1.14	0.87	1.49	0.340
Bang et al (2017)	Ipilimumab vs Best supportive care	0.90	0.50	1.60	0.721
Bellmunt et al (2017)	Pembrolizumab vs Chemotherapy	0.76	0.56	1.03	0.073
Carbone et al (2017)	Nivolumab vs Chemotherapy	1.04	0.77	1.41	0.799
Govindan et al (2017)	Ipilimumab+chemotherapy vs Placebo+chemotherapy	1.06	0.82	1.38	0.664
Kang et al (2017)	Nivolumab vs Placebo	0.53	0.38	0.74	0.000
Maio et al (2017)	Tremelimumab vs Placebo	0.99	0.77	1.27	0.936
Antonia et al (2018)	Durvalumab vs Placebo	0.76	0.55	1.06	0.101
Bang et al (2018)	Avelumab vs Chemotherapy	0.95	0.65	1.38	0.789
Barlesi et al (2018)	Avelumab vs Docetaxel	0.98	0.71	1.35	0.901
Fehrenbacher et al (2018)	Atezolizumab vs Docetaxel	0.75	0.61	0.92	0.005
Gandhi et al (2018)	Pembrolizumab+chemotherapy vs Placebo+chemotherapy	0.64	0.43	0.95	0.027
Horn et al (2018)	Atezolizumab+chemotherapy vs Placebo+chemotherapy	0.53	0.36	0.78	0.001
Larkin et al (2018)	Nivolumab vs Chemotherapy	0.62	0.41	0.94	0.024
Motzer et al (2018)	Nivolumab+ipilimumab vs Sunitinib	0.86	0.58	1.27	0.451
Paz-Ares et al (2018)	Pembrolizumab+chemotherapy vs Placebo+chemotherapy	0.74	0.51	1.07	0.111
Schmid et al (2018)	Atezolizumab+nab-paclitaxel vs Placebo+nab-paclitaxel	0.69	0.51	0.94	0.017
Shitara et al (2018)	Pembrolizumab vs Paclitaxel	0.90	0.63	1.29	0.564
Cohen et al (2019)	Pembrolizumab vs Standard of care	0.57	0.37	0.87	0.010
Eng et al (2019)	Atezolizumab+cobimetinib vs Regorafenib	1.50	0.85	2.64	0.161
Eng et al (2019)	Atezolizumab vs Regorafenib	0.99	0.47	2.08	0.979
Mok et al (2019)	Pembrolizumab vs Chemotherapy	0.82	0.66	1.01	0.067
Reck et al (2019)	Pembrolizumab vs Platinum-based chemotherapy	0.64	0.42	0.98	0.039
Reck et al (2019)	ABCP vs BCP	0.69	0.49	0.97	0.031
Reck et al (2019)	ACP vs BCP	0.97	0.71	1.32	0.847
Rini et al (2019)	Pembrolizumab+axitinib vs Sunitinib	0.59	0.36	0.97	0.037
West et al (2019)	Atezolizumab+carboplatin+nab-paclitaxel vs Chemotherapy	0.78	0.58	1.05	0.101
		0.78	0.72	0.84	0.000

Hazard ratio and 95% CI

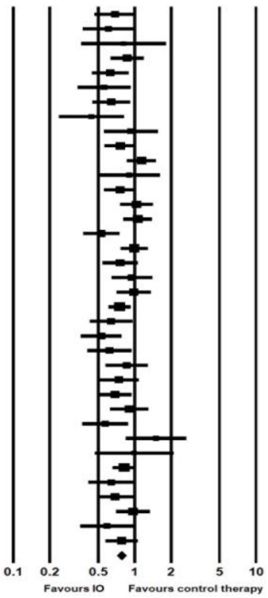

Overall survival for ≥65 years old

eFigure 4. Forest plots of interaction HRs for OS of immunotherapy vs control therapy by age.

Study-specific interaction HRs for each trial are represented by the squares, where the size of the square represents the weight of the trial in the meta-analysis and the horizontal line crossing the square represents the 95% CI. IO: immunotherapy; OS: overall survival; HRs: hazard ratios; CI: confidence interval; ABCP: atezolizumab + bevacizumab + carboplatin + paclitaxel; BCP: bevacizumab + carboplatin + paclitaxel; ACP: atezolizumab + carboplatin + paclitaxel.

Study name	Comparison	Statistics for each study: Hazard ratio	Lower limit	Upper limit	p-Value
Hodi et al (2010)	Ipilimumab+gp100 vs gp100	1.01	0.64	1.60	0.966
Hodi et al (2010)	Ipilimumab vs gp100	1.07	0.60	1.90	0.818
Robert et al (2011)	Ipilimumab+dacarbazine vs Placebo+dacarbazine	0.54	0.21	1.41	0.208
Ribas et al (2013)	Tremelimumab vs Standard of care chemotherapy	1.01	0.70	1.46	0.958
Borghaei et al (2015)	Nivolumab vs Docetaxel	1.29	0.84	1.98	0.242
Brahmer et al (2015)	Nivolumab vs Docetaxel	0.93	0.50	1.73	0.819
Motzer et al (2015)	Nivolumab vs Exerolimus	1.22	0.79	1.89	0.372
Robert et al (2015)	Nivolumab vs Dacarbazine	1.18	0.54	2.58	0.678
Ferris et al (2016)	Nivolumab vs Standard therapy	0.69	0.37	1.28	0.238
Herbst et al (2016)	Pembrolizumab vs Docetaxel	0.83	0.57	1.20	0.327
Reck et al (2016)	Ipilimumab+etoposide+platinum vs Placebo+etoposide+platinum	0.95	0.68	1.32	0.762
Bang et al (2017)	Ipilimumab vs Best supportive care	1.01	0.48	2.13	0.979
Bellmunt et al (2017)	Pembrolizumab vs Chemotherapy	0.99	0.63	1.55	0.965
Carbone et al (2017)	Nivolumab vs Chemotherapy	1.09	0.71	1.67	0.693
Govindan et al (2017)	Ipilimumab+chemotherapy vs Placebo+chemotherapy	0.77	0.54	1.10	0.155
Kang et al (2017)	Nivolumab vs Placebo	1.43	0.93	2.20	0.105
Maio et al (2017)	Tremelimumab vs Placebo	0.88	0.59	1.31	0.530
Antonia et al (2018)	Durvalumab vs Placebo	0.82	0.51	1.31	0.406
Bang et al (2018)	Avelumab vs Chemotherapy	1.28	0.79	2.08	0.320
Barlesi et al (2018)	Avelumab vs Docetaxel	0.86	0.56	1.32	0.491
Fehrenbacher et al (2018)	Atezolizumab vs Docetaxel	1.12	0.85	1.47	0.417
Gandhi et al (2018)	Pembrolizumab+chemotherapy vs Placebo+chemotherapy	0.67	0.40	1.13	0.131
Horn et al (2018)	Atezolizumab+chemotherapy vs Placebo+chemotherapy	1.74	1.03	2.93	0.038
Larkin et al (2018)	Nivolumab vs Chemotherapy	1.89	1.11	3.21	0.019
Motzer et al (2018)	Nivolumab+ipilimumab vs Sunitinib	0.62	0.38	1.01	0.053
Paz-Ares et al (2018)	Pembrolizumab+chemotherapy vs Placebo+chemotherapy	0.70	0.40	1.23	0.217
Schmid et al (2018)	Atezolizumab+nab-paclitaxel vs Placebo+nab-paclitaxel	1.22	0.85	1.75	0.277
Shitara et al (2018)	Pembrolizumab vs Paclitaxel	0.86	0.54	1.36	0.519
Cohen et al (2019)	Pembrolizumab vs Standard of care	1.65	1.01	2.70	0.046
Eng et al (2019)	Atezolizumab+cobimetinib vs Regorafenib	0.56	0.28	1.11	0.099
Eng et al (2019)	Atezolizumab vs Regorafenib	1.21	0.52	2.82	0.659
Mok et al (2019)	Pembrolizumab vs Chemotherapy	0.99	0.74	1.32	0.945
Reck et al (2019)	Pembrolizumab vs Platinum-based chemotherapy	0.94	0.50	1.76	0.847
Reck et al (2019)	ABCP vs BCP	1.13	0.74	1.72	0.570
Reck et al (2019)	ACP vs BCP	0.78	0.52	1.17	0.230
Rini et al (2019)	Pembrolizumab+axitinib vs Sunitinib	0.80	0.41	1.56	0.511
West et al (2019)	Atezolizumab+carboplatin+nab-paclitaxel vs Chemotherapy	1.01	0.66	1.55	0.964
		1.00	0.92	1.08	0.944

Hazard ratio and 95% CI

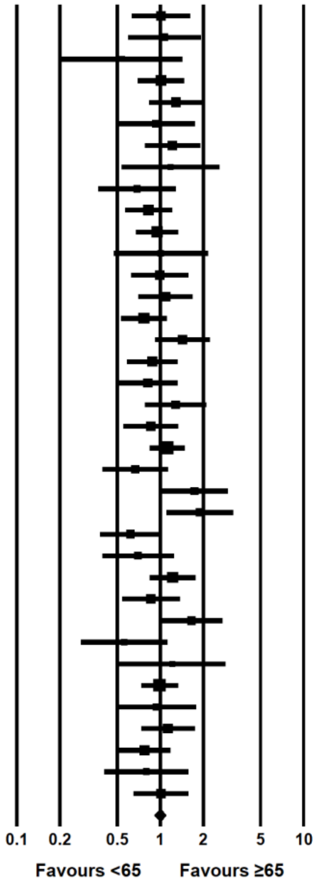

Overall survival for <65 vs ≥65 years old

© 2020 Yang F et al. *JAMA Network Open.*

eFigure 5. Forest plots of HRs for OS of immunotherapy vs control therapy in (A) ECOG PS 0 and (B) ECOG PS ≥1 patients.

IO: immunotherapy; OS: overall survival; HRs: hazard ratios; CI: confidence interval; ABCP: atezolizumab + bevacizumab + carboplatin + paclitaxel; BCP: bevacizumab + carboplatin + paclitaxel; ACP: atezolizumab + carboplatin + paclitaxel; ECOG PS: Eastern Cooperative Oncology Group Performance Status.

A

Study name	Comparison	Hazard ratio	Lower limit	Upper limit	p-Value
		Statistics for each study			
Robert et al (2011)	Ipilimumab+dacarbazine vs Placebo+dacarbazine	0.54	0.32	0.93	0.025
Kwon et al (2014)	Ipilimumab vs Placebo	0.72	0.55	0.94	0.016
Borghaei et al (2015)	Nivolumab vs Docetaxel	0.64	0.44	0.93	0.019
Brahmer et al (2015)	Nivolumab vs Docetaxel	0.48	0.24	0.97	0.042
Robert et al (2015)	Nivolumab vs Dacarbazine	0.32	0.20	0.52	0.000
Ferris et al (2016)	Nivolumab vs Standard therapy	0.60	0.30	1.21	0.156
Herbst et al (2016)	Pembrolizumab vs Docetaxel	0.73	0.52	1.02	0.067
Reck et al (2016)	Ipilimumab+etoposide+platinum vs Placebo+etoposide+platinum	1.28	0.97	1.68	0.076
Bang et al (2017)	Ipilimumab vs Best supportive care	1.22	0.70	2.12	0.482
Beer et al (2017)	Ipilimumab vs Placebo	1.09	0.85	1.40	0.498
Carbone et al (2017)	Nivolumab vs Chemotherapy	1.11	0.74	1.66	0.613
Govindan et al (2017)	Ipilimumab+chemotherapy vs Placebo+chemotherapy	0.99	0.73	1.34	0.948
Kang et al (2017)	Nivolumab vs Placebo	0.59	0.40	0.87	0.008
Antonia et al (2018)	Durvalumab vs Placebo	0.82	0.57	1.17	0.274
Bang et al (2018)	Avelumab vs Chemotherapy	1.48	0.97	2.26	0.071
Barlesi et al (2018)	Avelumab vs Docetaxel	0.73	0.50	1.07	0.109
Fehrenbacher et al (2018)	Atezolizumab vs Docetaxel	0.80	0.63	1.02	0.069
Gandhi et al (2018)	Pembrolizumab+chemotherapy vs Placebo+chemotherapy	0.44	0.28	0.70	0.001
Horn et al (2018)	Atezolizumab+chemotherapy vs Placebo+chemotherapy	0.79	0.49	1.27	0.332
Larkin et al (2018)	Nivolumab vs Chemotherapy	0.95	0.67	1.34	0.772
Paz-Ares et al (2018)	Pembrolizumab+chemotherapy vs Placebo+chemotherapy	0.54	0.29	0.99	0.047
Powles et al (2018)	Atezolizumab vs Chemotherapy	0.84	0.66	1.06	0.149
Schmid et al (2018)	Atezolizumab+nab-paclitaxel vs Placebo+nab-paclitaxel	0.78	0.64	0.95	0.011
Shitara et al (2018)	Pembrolizumab vs Paclitaxel	0.69	0.49	0.97	0.033
Cohen et al (2019)	Pembrolizumab vs Standard of care	0.87	0.57	1.32	0.516
Eng et al (2019)	Atezolizumab+cobimetinib vs Regorafenib	1.13	0.71	1.81	0.611
Eng et al (2019)	Atezolizumab vs Regorafenib	1.65	0.96	2.82	0.068
Mok et al (2019)	Pembrolizumab vs Chemotherapy	0.77	0.57	1.04	0.084
Reck et al (2019)	Pembrolizumab vs Platinum-based chemotherapy	0.78	0.44	1.38	0.391
Reck et al (2019)	ABCP vs BCP	0.75	0.53	1.07	0.108
Reck et al (2019)	ACP vs BCP	0.85	0.61	1.18	0.334
West et al (2019)	Atezolizumab+carboplatin+nab-paclitaxel vs Chemotherapy	0.85	0.59	1.22	0.381
		0.81	0.73	0.90	0.000

Hazard ratio and 95% CI

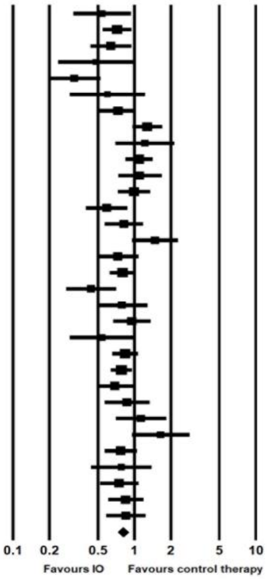

Overall survival for ECOG PS 0

B

Study name	Comparison	Hazard ratio	Lower limit	Upper limit	p-Value
		Statistics for each study			
Robert et al (2011)	Ipilimumab+dacarbazine vs Placebo+dacarbazine	0.47	0.21	1.04	0.061
Kwon et al (2014)	Ipilimumab vs Placebo	0.94	0.76	1.16	0.566
Borghaei et al (2015)	Nivolumab vs Docetaxel	0.80	0.63	1.01	0.058
Brahmer et al (2015)	Nivolumab vs Docetaxel	0.54	0.39	0.74	0.000
Robert et al (2015)	Nivolumab vs Dacarbazine	0.64	0.40	1.03	0.067
Ferris et al (2016)	Nivolumab vs Standard therapy	0.71	0.53	0.96	0.024
Herbst et al (2016)	Pembrolizumab vs Docetaxel	0.63	0.51	0.78	0.000
Reck et al (2016)	Ipilimumab+etoposide+platinum vs Placebo+etoposide+platinum	0.99	0.83	1.18	0.911
Bang et al (2017)	Ipilimumab vs Best supportive care	0.69	0.41	1.16	0.162
Beer et al (2017)	Ipilimumab vs Placebo	1.08	0.72	1.62	0.710
Carbone et al (2017)	Nivolumab vs Chemotherapy	1.02	0.79	1.32	0.880
Govindan et al (2017)	Ipilimumab+chemotherapy vs Placebo+chemotherapy	0.86	0.70	1.05	0.145
Kang et al (2017)	Nivolumab vs Placebo	0.68	0.53	0.87	0.002
Antonia et al (2018)	Durvalumab vs Placebo	0.58	0.42	0.80	0.001
Bang et al (2018)	Avelumab vs Chemotherapy	1.01	0.76	1.35	0.946
Barlesi et al (2018)	Avelumab vs Docetaxel	0.99	0.77	1.28	0.938
Fehrenbacher et al (2018)	Atezolizumab vs Docetaxel	0.77	0.65	0.91	0.002
Gandhi et al (2018)	Pembrolizumab+chemotherapy vs Placebo+chemotherapy	0.53	0.39	0.73	0.000
Horn et al (2018)	Atezolizumab+chemotherapy vs Placebo+chemotherapy	0.68	0.50	0.93	0.015
Larkin et al (2018)	Nivolumab vs Chemotherapy	0.89	0.60	1.32	0.559
Paz-Ares et al (2018)	Pembrolizumab+chemotherapy vs Placebo+chemotherapy	0.66	0.48	0.90	0.010
Powles et al (2018)	Atezolizumab vs Chemotherapy	0.87	0.72	1.06	0.163
Schmid et al (2018)	Atezolizumab+nab-paclitaxel vs Placebo+nab-paclitaxel	0.82	0.66	1.02	0.080
Shitara et al (2018)	Pembrolizumab vs Paclitaxel	0.98	0.73	1.32	0.894
Cohen et al (2019)	Pembrolizumab vs Standard of care	0.78	0.62	0.98	0.033
Eng et al (2019)	Atezolizumab+cobimetinib vs Regorafenib	0.85	0.56	1.30	0.449
Eng et al (2019)	Atezolizumab vs Regorafenib	0.85	0.52	1.38	0.511
Mok et al (2019)	Pembrolizumab vs Chemotherapy	0.83	0.71	0.98	0.023
Reck et al (2019)	Pembrolizumab vs Platinum-based chemotherapy	0.56	0.39	0.81	0.002
Reck et al (2019)	ABCP vs BCP	0.75	0.59	0.95	0.015
Reck et al (2019)	ACP vs BCP	0.84	0.67	1.06	0.136
West et al (2019)	Atezolizumab+carboplatin+nab-paclitaxel vs Chemotherapy	0.77	0.59	1.01	0.060
		0.79	0.74	0.84	0.000

Hazard ratio and 95% CI

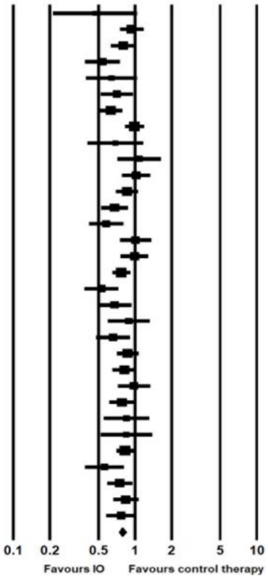

Overall survival for ECOG PS ≥1

© 2020 Yang F et al. *JAMA Network Open.*

eFigure 6. Forest plots of interaction HRs for OS of immunotherapy vs control therapy by ECOG PS.

Study-specific interaction HRs for each trial are represented by the squares, where the size of the square represents the weight of the trial in the meta-analysis and the horizontal line crossing the square represents the 95% CI. IO: immunotherapy; OS: overall survival; HRs: hazard ratios; CI: confidence interval; ABCP: atezolizumab + bevacizumab + carboplatin + paclitaxel; BCP: bevacizumab + carboplatin + paclitaxel; ACP: atezolizumab + carboplatin + paclitaxel; ECOG PS: Eastern Cooperative Oncology Group Performance Status.

Study name	Comparison	Statistics for each study			
		Hazard ratio	Lower limit	Upper limit	p-Value
Robert et al (2011)	Ipilimumab+dacarbazine vs Placebo+dacarbazine	1.15	0.44	3.00	0.775
Kwon et al (2014)	Ipilimumab vs Placebo	0.77	0.54	1.09	0.139
Borghaei et al (2015)	Nivolumab vs Docetaxel	0.80	0.52	1.24	0.314
Brahmer et al (2015)	Nivolumab vs Docetaxel	0.89	0.41	1.93	0.768
Robert et al (2015)	Nivolumab vs Dacarbazine	0.50	0.25	0.99	0.048
Ferris et al (2016)	Nivolumab vs Standard therapy	0.85	0.39	1.84	0.679
Herbst et al (2016)	Pembrolizumab vs Docetaxel	1.16	0.78	1.73	0.465
Reck et al (2016)	Ipilimumab+etoposide+platinum vs Placebo+etoposide+platinum	1.29	0.93	1.79	0.127
Bang et al (2017)	Ipilimumab vs Best supportive care	1.77	0.83	3.78	0.140
Beer et al (2017)	Ipilimumab vs Placebo	1.01	0.63	1.62	0.967
Carbone et al (2017)	Nivolumab vs Chemotherapy	1.09	0.67	1.77	0.727
Govindan et al (2017)	Ipilimumab+chemotherapy vs Placebo+chemotherapy	1.15	0.80	1.65	0.449
Kang et al (2017)	Nivolumab vs Placebo	0.87	0.55	1.38	0.553
Antonia et al (2018)	Durvalumab vs Placebo	1.41	0.88	2.26	0.155
Bang et al (2018)	Avelumab vs Chemotherapy	1.47	0.88	2.45	0.140
Barlesi et al (2018)	Avelumab vs Docetaxel	0.74	0.46	1.18	0.206
Fehrenbacher et al (2018)	Atezolizumab vs Docetaxel	1.04	0.78	1.39	0.790
Gandhi et al (2018)	Pembrolizumab+chemotherapy vs Placebo+chemotherapy	0.83	0.47	1.46	0.517
Horn et al (2018)	Atezolizumab+chemotherapy vs Placebo+chemotherapy	1.16	0.66	2.04	0.608
Larkin et al (2018)	Nivolumab vs Chemotherapy	1.07	0.63	1.81	0.801
Paz-Ares et al (2018)	Pembrolizumab+chemotherapy vs Placebo+chemotherapy	0.82	0.41	1.63	0.571
Powles et al (2018)	Atezolizumab vs Chemotherapy	0.97	0.71	1.32	0.845
Schmid et al (2018)	Atezolizumab+nab-paclitaxel vs Placebo+nab-paclitaxel	0.95	0.71	1.28	0.733
Shitara et al (2018)	Pembrolizumab vs Paclitaxel	0.70	0.45	1.10	0.121
Cohen et al (2019)	Pembrolizumab vs Standard of care	1.12	0.69	1.81	0.643
Eng et al (2019)	Atezolizumab+cobimetinib vs Regorafenib	1.33	0.71	2.50	0.375
Eng et al (2019)	Atezolizumab vs Regorafenib	1.94	0.94	4.00	0.073
Mok et al (2019)	Pembrolizumab vs Chemotherapy	0.93	0.67	1.29	0.664
Reck et al (2019)	Pembrolizumab vs Platinum-based chemotherapy	1.39	0.71	2.73	0.339
Reck et al (2019)	ABCP vs BCP	1.00	0.66	1.52	1.000
Reck et al (2019)	ACP vs BCP	1.01	0.68	1.51	0.961
West et al (2019)	Atezolizumab+carboplatin+nab-paclitaxel vs Chemotherapy	1.10	0.70	1.73	0.682
		1.01	0.94	1.10	0.738

Hazard ratio and 95% CI

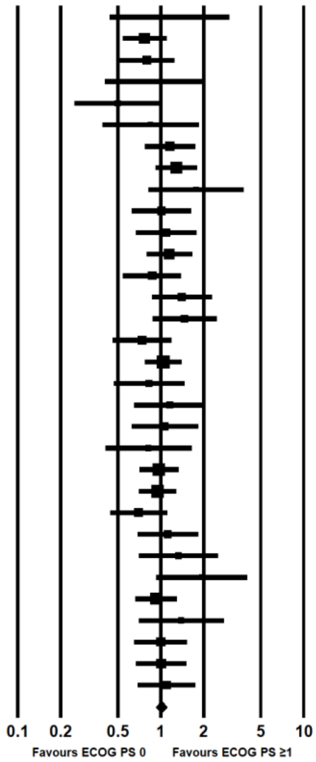

Overall survival for ECOG PS 0 vs ≥1

© 2020 Yang F et al. *JAMA Network Open.*
